# Cointegration as a mechanism for the evolution of a KPC-producing multidrug resistance plasmid in *Proteus mirabilis*

**DOI:** 10.1080/22221751.2020.1773322

**Published:** 2020-06-04

**Authors:** Xiaoting Hua, Linyue Zhang, Robert A. Moran, Qingye Xu, Long Sun, Willem van Schaik, Yunsong Yu

**Affiliations:** a Department of Infectious Diseases, Sir Run Run Shaw Hospital, College of Medicine, Zhejiang University, Hangzhou, People’s Republic of China; b Key Laboratory of Microbial Technology and Bioinformatics of Zhejiang Province, Hangzhou, People’s Republic of China; c Department of Clinical Laboratory, Hangzhou Women’ s Hospital (Hangzhou Maternity and Child Health Care Hospital), Hangzhou, People’s Republic of China; d Institute of Microbiology and Infection, College of Medical and Dental Sciences, The University of Birmingham, Birmingham, United Kingdom; e State Key Laboratory for Diagnosis and Treatment of Infectious Diseases, Collaborative Innovation Center for Diagnosis and Treatment of Infectious Diseases, The First Affiliated Hospital, College of Medicine, Zhejiang University, Hangzhou, People’s Republic of China; f Department of Clinical Laboratory, Hangzhou Hospital of Zhejiang Provincial Corps, Chinese People’s Armed Police Forces, Hangzhou, People’s Republic of China

**Keywords:** *Proteus mirabilis*, plasmids, evolution, *bla*_KPC-2_, IS*26*

## Abstract

The incidence and transmission of *Klebsiella pneumoniae* carbapenemase (KPC) producing plasmids have been well documented. However, the evolutionary dynamics of KPC plasmids and their fitness costs are not well characterized. Here, two carbapenemase-producing plasmids from *Proteus mirabilis*, pT18 and pT211 (both carrying *bla*_KPC-2_), were characterized through whole genome sequencing. pT211 is a 24.2 kbp N-type plasmid that contains *bla*_KPC-2_ and a single copy of the IS*6*-family insertion sequence IS*26*. pT18 is a 59 kbp cointegrate plasmid comprised of sequences derived from three different plasmids: a close relative of pT211 (containing *bla*_KPC-2_), an FII-33 plasmid (*bla*_TEM-1B_, *bla*_CTX-M-65_, *rmtB* and *fosA3*) and a rolling-circle plasmid. The segments of pT18 derived from each of the different plasmids are separated by copies of IS*26*, and sequence analysis indicated that pT18 was likely generated by both conservative and replicative IS*26*-mediated cointegrate formation. pT18 and pT211 were transferred into *Escherichia coli* DH5α separately to assess the impact of plasmids on host fitness. Only DH5α harbouring pT18 grew slower than the wild type in antibiotic-free media. However, in sub-inhibitory concentrations of fosfomycin and amikacin, cells containing pT18 grew faster than the wild type, and the minimum concentrations of fosfomycin and amikacin required to observe an advantage for plasmid-carrying cells were 1/3 and 1/20 the DH5α MIC, respectively. This study highlights the importance of the role of cointegrate plasmids in the dissemination of antibiotic resistance genes between pathogenic bacterial species, and highlights the importance of sub-inhibitory concentrations of antibiotics to the persistence of such plasmids.

## Introduction

The rapid emergence of carbapenem resistance in Gram-negative bacteria is a major public health problem. The *Klebsiella pneumoniae* carbapenemase (KPC) enzyme hydrolyzes most β-lactam antimicrobial agents, including carbapenems, and the global spread of *bla*_KPC_ genes has been well documented. KPC-producing bacteria, usually belonging to the order Enterobacterales, have been detected across the planet, and several KPC variants have been described [1].

*Proteus mirabilis* is a member of the Enterobacterales, and its unique swarming motility aids in colonizing the human urinary tract, making it a leading cause of urinary tract infections [2]. The first report of KPC carriage in *P. mirabilis* was KPC-2 in a bloodstream isolate in 2008 [3]. The clonal dissemination of KPC-2-producing *P. mirabilis* in Intensive Care Units (ICUs) in China was reported in 2012, when 19 carbapenem-resistant *P. mirabilis* isolates harboured *bla*_KPC-2_ in a genetic environment (ISKpn8-*bla*_KPC-2_-ISKpn6-like) that was identical to a region of the previously described FII-K plasmid pKP048 from *K. pneumoniae* [4]. Our previous study showed that in the majority of Chinese ICU *P. mirabilis* isolates, the *bla*_KPC-2_ gene was primarily located on plasmids of either 26 kbp or 55 kbp [5].

Plasmids play important roles in bacterial physiology. In clinical settings, conjugative plasmids are the main contributors to horizontal gene transfer (HGT), mediating the direct exchange of multiple resistance genes that help bacteria rapidly adapt to clinical environments [6,7]. While not all plasmids are self-transmissible [8], recent evidence suggests that a greater proportion of plasmids than previously appreciated might be capable of HGT via mobilization [9]. Mobile genetic elements (MGEs), particularly insertion sequence (IS) elements, can reorganize plasmids, and IS*26* plays a particularly important role in the dissemination of antibiotic resistance genes in Gram-negative bacteria [10,11]. An important property of IS*26* is its ability to generate cointegrate DNA molecules comprised of two or more previously distinct DNA elements [11,12]. While cointegrates can occur via recombination between homologous DNA segments at low frequency [13], IS*26* can generate cointegrates via replicative or high-frequency conservative mechanisms [12]. It has also been demonstrated that once a chromosome or plasmid possesses a copy of IS*26*, it is predisposed to acquire additional IS*26* translocatable units (TUs) [14]. IS*26* can also cause deletions of adjacent DNA segments [15,16] and these events can complicate comparative analyses, particularly when target site duplications or regions flanking integrated sequences are lost.

The importance of cointegrate plasmids for the spread of multiple antibiotic resistance genes among Enterobacterales is well-documented [17]. A previous study showed that a plasmid that carried *bla*_OXA-427_ in *K. pneumoniae* was integrated into a FIB plasmid to form a megaplasmid with multiple MDR regions [18]. A KPC-carrying plasmid in *E. coli*, pBK32533, is an IS*26*-generated cointegrate plasmid containing pBK30661 and an additional 170 kbp genetic element [19]. Plasmid cointegrations were also reported previously for carbapenemase-producing Enterobacterales [19]. However, to our knowledge, plasmid cointegration in *P. mirabilis* has only been reported once before [20] and hybrid plasmids containing *bla*_KPC-2_ have not yet been reported in *P. mirabilis*.

In this study, we sequenced the complete genomes of two KPC-2-producing multidrug-resistant *P. mirabilis* isolates from different patients in the same ward of a rehabilitation department in a teaching hospital in Hangzhou, China. Strain T21 was isolated from sputum in 2013 and strain T18 was isolated from urine in 2014. The antibiotic resistance gene and plasmid content of each isolate was characterized, and the isolates’ plasmids were examined in detail to provide insights into the evolution of plasmids carrying *bla*_KPC-2_ in *P. mirabilis*. The fitness costs of each *bla*_KPC-2_-bearing plasmid was determined in *P. mirabilis* and *E. coli*, and the sub-inhibitory concentrations of antibiotics required to select for plasmid-containing cells was determined via competition experiments with plasmid-free cells. The distribution of *bla*_KPC-2_-containing plasmids in a collection of 21 *Pmirabilis* isolated in the same hospital at the same time as T21 and T18 was examined via Illumina sequencing and mapping to complete plasmid sequences.

## Materials and methods

### Bacterial strains and media

*P. mirabilis* T21 was isolated from the sputum of a 63-year-old male with a pulmonary infection in June 2013. *P. mirabilis* T18 was isolated from the urine of a 49-year-old female with a pulmonary infection in January 2014. The isolates were collected from patients in the same ward in a teaching hospital in Hangzhou, China. A further 21 clinical *P. mirabilis* isolates collected from the same teaching hospital between (2013 and 2014) were also included in this study. These strains were identified as *P. mirabilis* using conventional biochemical tests and confirmed by 16S rRNA gene sequencing analysis. The plasmid in *P. mirabilis* T18 was isolated and electroporated into *E. coli* DH5α, and the resulting strain XH1096 (DH5α pT18) was used for subsequent experiments. Plasmid pT211 was also electroporated into *E. coli* DH5α, and the resulting strain was named XH1097 (DH5α pT211). pT211 and pT18 were electroporated into the antibiotic-sensitive *P. mirabilis* strain XH1568, generating XH1570 (XH1568 pT211) and XH1571 (XH1568 pT18). XH1568 was isolated from a bile sample of a 76-year-old male with gallbladder carcinoma in Sir Run Run Shaw Hospital, Hangzhou in April 2019. All strains used in this study are listed in Table S1. Relevant patient clinical information for strains T18 and T21 is listed in Table S2. Oligonucleotide primers used in this study are listed in Table S3. The liquid growth medium used in this study was Mueller Hinton (MH) Broth (Oxoid, UK).

### MIC measurements

MIC assays for fosfomycin were performed by agar dilution or Etest (Bestbion GmbH, Liofilchem, Italy), and MICs for amikacin were determined by broth microdilution or Etest (AB bioMerieux, Solna, Sweden) or the broth microdilution method. The results of susceptibility testing were interpreted according to the Clinical and Laboratory Standards Institute guidelines. A quality control strain (*E. coli* ATCC 25922) was included in all MIC assays.

### Genomic DNA sequencing

Genomic DNA was isolated from the strains using the QIAamp DNA Minikit (Qiagen, Valencia, CA) according to the manufacturer’s protocol. For T18 and T21, the chromosome and plasmids were sequenced by the PacBio RS platform (Pacific Biosciences, Menlo Park, CA) after library construction. *De novo* assembly of the reads was performed using continuous long reads following the Hierarchical Genome Assembly Process (HGAP) workflow (PacBio DevNet; Pacific Biosciences, Menlo Park, CA) as available in SMRT Analysis v2.3.0. For XH1568, Long-read library preparation for Nanopore sequencing was performed with a 1D sequencing kit (SQK-LSK109; Nanopore) without fragmentation. The libraries were then sequenced on a MinION device with a 1D flow cell (FlO-MIN106; Nanopore) and base called with Guppy v2.3.5 (Nanopore). The long read and short read sequence data were used in a hybrid *de novo* assembly using Unicycler v0.4.8 [21], then polished by Pilon v1.23 [22]. Annotation of the assemblies was performed using the NCBI PGAP annotation pipeline [23] and checked manually.

### Phylogenomic and coverage analysis

The raw data from *P. mirabilis* T18 and the 21 additional clinical isolates were mapped to the T21 genome using Snippy v4.4.5 (https://github.com/tseemann/snippy). Single-nucleotide polymorphisms (SNPs) were called using default parameters. The core-genome SNPs obtained were aligned for all isolates to construct a phylogeny. The phylogenetic tree was constructed by the maximum-likelihood method using FastTree v2.1.10 [24], which was run using the generalized time-reversible (GTR) model of nucleotide evolution and incorporated the Gamma model for rate heterogeneity. Antimicrobial resistance genes were identified using ABRicate version 0.9.8 with the ncbi database (https://github.com/tsee-mann/abricate). Plasmid coverage was identified by mapping Illumina reads using BWA 0.7.17-r1188, followed by the analysis of sequencing depth by SAMtools. The comparison of genome sequences of the 21 *Pmirabilis* isolates, and pT18 and pT211 was performed by the BLAST Ring Image Generator (BRIG) [25]. The phylogenetic tree was visualized in R (https://www.r-project.org) by using the package ggtree [26].

### Conjugation assays

To test the transferability of pT211, pT212 and pT18, T21 and T18 were used as the donors for conjugation assays. Sodium azide-resistant *E. coli* J53 was used as the recipient strain. The *E. coli* J53 transconjugants were selected on Mueller-Hinton agar medium supplemented with sodium azide (150 mg/L) plus amikacin (100 mg/L) for pT212 and pT18 conjugation experiments and sodium azide (150 mg/L) plus ampicillin (100 mg/L) for pT211 conjugation experiments. Transconjugants were screened for the presence of pT211, pT212and pT18 by PCR and Sanger sequencing. The species identity of each transconjugant was confirmed by 16S rRNA gene sequencing. By counting the number of transconjugants on Mueller-Hinton agar medium supplemented with sodium azide (150 mg/L) plus amikacin (100 mg/L) and the number of recipients on Mueller-Hinton agar with sodium azide (150 mg/L). The conjugation frequency was calculated as the number of transconjugants per recipient.

### Growth rate determination

Four independent cultures per strain were grown overnight, diluted 1:1000 in MH broth (with antibiotic when indicated) and aliquoted into a flat-bottom honeycomb 100-well plate in three replicates. When testing the growth rate in the presence of fosfomycin, MH broth containing 25 mg/L glucose 6-phosphate was used. The plate was incubated at 37 °C with agitation. The OD_600_ of each culture was determined every 5 min for 16 h using a Bioscreen C MBR machine (Oy Growth Curves Ab Ltd., Finland). The growth rate was estimated based on OD_600_ curves using an R script, as previously described [27].

### Determination of minimal selective concentrations

To determine the minimal selective concentrations (MSCs) of fosfomycin and amikacin, we competed *E. coli* DH5α with the plasmid pT18 (XH1096) against *E. coli* XH141 (DH5α carrying pEL-polB-sYFP2). The plasmid pEL-polB-sYPF2 was generated using Gibson cloning by fusing the *polB* promoter to the gene encoding sYPF2 and subsequent insertion of the construct into pEL3A15-B0034-SYFP2. The *polB* promoter was amplified from *Salmonella enterica* serovar Typhimurium LT2 (genome position: 115416 - 115802 bp) with primers polB_F and polB_R (Table S3). The MSC was considered as a measure of the fitness cost of the resistance plasmid balanced by antibiotic resistance. Fosfomycin and amikacin were chosen as representative drugs for the resistance genes *fosA3* and *rmtB* carried by pT18. We used flow cytometry to distinguish the two *E. coli* populations that were either non-fluorescent (DH5α pT18) or tagged with the yellow fluorescent SYFP2 protein (XH141). To assess the cost of the SYFP2 plasmid in DH5α, control experiments were performed between wild type and DH5α carrying the SYFP2-encoding plasmid. The stability of the SYFP2-encoding plasmid without antibiotics was also assessed. For all competition experiments, the selection coefficient was corrected using the fitness cost caused by the plasmid harbouring fluorescent proteins.

## Results

### Characterization of *P. mirabilis* T18 and T21

The complete genome sequences of *P. mirabilis* T18 and T21 were assembled. T18 has a 4,131,426 bp chromosome and carries a single plasmid, pT18 (Table 1). T21 has a 4,090,879 bp chromosome and carries two plasmids, pT211 and pT212 (Table 1). In both isolates, the antibiotic resistance genes *bla*_TEM-1B_ (conferring resistance to ampicillin), *strAB* (streptomycin), *aadA1* and *aadA5* (streptomycin and spectinomycin), *aphA1a* (kanamycin and neomycin), *aac(3)-IId* (gentamicin and tobramycin), *sat2* (streptothricin), *dfrA1* and *dfrA17* (trimethoprim), *sul1* and *sul2* (sulphonamides) and *catA1* (chloramphenicol) are located in the same structures in the chromosome. The *dfrA1*-*sat2*-*aadA1* gene cassettes in both strains were found in the class 2 integron of the well-studied transposon Tn*7* [28], which was inserted in the *attTn7* site 25 bp downstream of the *glmS* gene and flanked by the 5 bp target site duplication (TSD) CCAAT.

**Table 1.** Resistance genes in the P. mirabilis genome and associated plasmid**s.**

Strain	Genome size (bp)	Resistance genes (chromosome)	Plasmid (size in bp)	Plasmid type	Resistance genes (plasmid)
T18	4,131,426	*aphA1a*, *strB*, *strA*, *aac(3)-IId*, *aadA5*, *aadA1*, *sul1*, *dfrA1*, *dfrA17*, *sat2*, *bla*_TEM-1B_	pT18 (59,035)	N FII-33 Rolling-circle	*bla*_KPC-2_, *bla*_CTX-M-65_, *bla*_TEM-1B_, *rmtB*, *fosA3*
T21	4,090,879	*aphA1a*, *strB*, *strA*, *aac(3)-IId*, *aadA5*, *aadA1*, *sul1*, *dfrA1*, *dfrA17 sat2*, *bla*_TEM-1B_	pT211 (24,225)	N	*bla*_KPC-2_
			pT212 (171,489)	C2	*aac(3)-IId*, *aadA2*, *aphA1c*, *rmtB*, *strA*, *strB*, *bla*_CTX-M-14_, *bla*_TEM-1B_, *sul1*, *sul2*, *tet*(G), *dfrA12*

**Figure 1.**
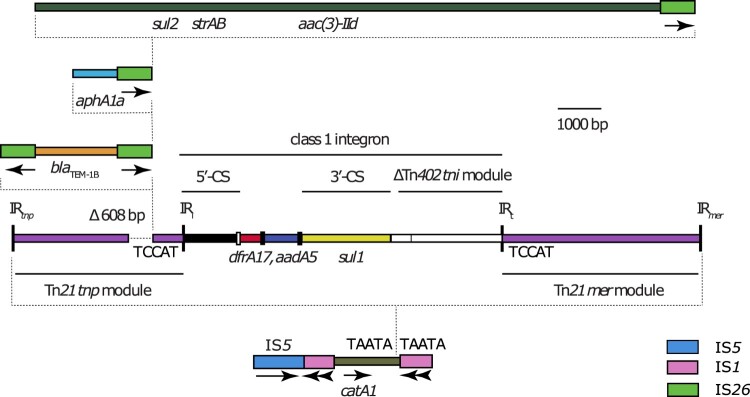
Structure of the chromosomal antibiotic resistance gene region in T18 and T21. Scaled, linear diagram of the multidrug resistance region found in the chromosomes of T18 and T21. DNA sequences of different origin are drawn as coloured boxes. Thicker boxes represent insertions sequences IS*26* (green), IS*1* (pink) and IS*5* (blue), with the direction of their transposase genes indicated by arrows beneath. Sequences derived from Tn*21* are purple, with the transposition (*tnp*) and mercury resistance (*mer*) modules labelled. The extent of the class 1 integron is shown by a labelled horizontal line, with the 5’-conserved segment (5’-CS), 3’-conserved segment (3’-CS) and truncated Tn*402* transposition (*tni*) module indicated by labelled horizontal lines. The *attI* site is shown as a small, open box, and the *attC* sites of gene cassettes are shown as small, filled boxes. The locations of inverted repeat (IR) sequences are indicated by labelled, vertical lines. The location of the 608 bp deletion in the Tn*21 tnp* module is labelled above the box that represents the sequence, and the positions of antibiotic resistance genes are indicated by labels below. Dotted lines are used to denote discrete elements within the resistance island and the position in which they have inserted in the sequence shown below them. If present, target site duplication sequences generated by insertions are shown either side of the vertical dotted lines that indicate insertion position, or adjacent to inverted repeats IR_i_ and IR_t_. Drawn to scale from GenBank accession CP017085.

The remainder of the chromosomal antibiotic resistance genes were located 79,865 bp away from the *tns* end of Tn*7*, in a complex region derived from Tn*2670* [29]. The complex region is bounded by directly oriented copies of IS*1*, but the IS*1* on the left has been truncated by a copy of IS*5* (Figure 1). As in Tn*2670*, the *catA1*-containing passenger segment between the copies of IS*1* includes a Tn*21*-like transposon, flanked by the 5 bp TSD TAATA, which has a class 1 integron in the same position as the class 1 integron in Tn*21* (In2), flanked by the 5 bp TSD TCCAT (Figure 1). The class 1 integron in T18 and T21 contains a different cassette array (*dfrA17*-*aadA5*) to the one found in In2 (*aadA1*), and does not include the IS*1353* and IS*1326* that are inserted between the 3’-conserved segment (yellow in Figure 1) and the remnant of the Tn*402 tni* module (white with black stripes in Figure 1) in In2.

In T18 and T21, the 4,594 bp mercuric chloride resistance (*mer*) module of Tn*21* is complete and uninterrupted. However, at the left end the Tn*21* transposition (*tnp*) module has been interrupted by a 21,003 bp region bounded at both ends by copies of IS*26*. Relative to Tn*21* (GenBank accession AF071413), an IS*26* in T18 and T21 has deleted 608 bp of the transposition module (Figure 1), including the final 308 bp of the *tnpR* gene and the first 298 bp of the *tnpA* gene, rendering the transposon immobile. The 21,003 bp region contains four copies of IS*26*, and at least one antibiotic resistance gene lies between each pair of IS; *bla*_TEM-1B_, *aphA1c*, or *sul2*-*strAB*-*aac(3)-IId* (Figure 1). Three of these IS*26* are in direct orientation (Figure 1), and it is likely that the individual segments between pairs of IS*26* were acquired as one or more TUs [11] that inserted into the resistance region via conservative transposition.

### The *P. mirabilis* T21 plasmid pT212

A further 13 antibiotic resistance genes in T21 are found in its two plasmids. While some of these genes are also located in the chromosome, the plasmid-borne resistance gene complement confers resistance to a range of antibiotic classes.

The largest plasmid in T21, pT212, is 171,489 bp, has an average G + C content of 52.2%, and contains a total of 201 ORFs. The replicon of pT212 is identical to that of the reference IncC plasmid R55 (GenBank accession JQ010984), which was recovered from a *K. pneumoniae* isolated in France in 1969 [30]. IncC plasmids have been reviewed recently, and a method for subtyping them as IncC type 1 or IncC type 2 was devised [31]. In accordance with that method, the plasmid backbone regions R1 (5,541 bp), R2 (5,161 bp), i1 (628 bp) and i2 (662 bp) of pT212 were compared to the corresponding regions of R55. Segments R2, i1 and i2 of pT212 were identical to those of R55, and R1 differed by just one nucleotide. Thus, pT212 is a type 2 IncC plasmid.

Comparison of the complete sequence of pT212 to that of R55 revealed that a large segment of the pT212 backbone has been inverted (Figure S1A). Close analysis of the sequences flanking mobile elements in pT212 revealed that the inversion occurred between two, inversely oriented copies of IS*903*, and reversing the inversion *in silico* restored flanking TSDs around two elements (Figure S1B). This allowed for characterization of the acquired antibiotic resistance gene regions (Figure S1C).

In a variant of the well-characterized ARI-B resistance island, located upstream of the *parAB* genes and derived from the element GI*sul2* [31], pT212 contains the resistance genes *bla*_TEM-1B_, *strAB*, *aphA1c*, *aac(3)-IId*, *aadA2* (streptomycin and spectinomycin resistance), *tet*(G) (tetracycline), *sul2* (sulphonamides), *dfrA12* (trimethoprim), *rmtB* (all clinically relevant aminoglycosides) and a truncated copy of *sul1* (Figure S1C). The final resistance gene in pT212, *bla*_CTX-M-14_ (cephalosporin resistance), is in a different region of the backbone. It is part of a 1,476 bp passenger segment in a composite transposon comprised of inversely-oriented copies of IS*903*. That transposon is inside a copy of Tn*1722*, which has inserted into the 3’ end of the backbone gene *ter*, generating the 5 bp TSD CTGGA (Figure S1C), in a position which has been called resistance island 9 in the IncC backbone [31].

### The *P. mirabilis* T21 plasmid pT211

**Figure 2.**
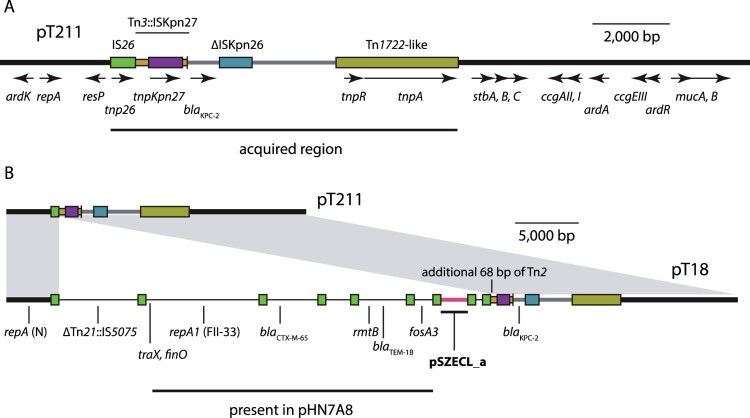
KPC-2-determining plasmids in T21 and T18. A) Scaled, linear diagram of N-type plasmid pT211 from T21. The plasmid backbone is shown as a thick, black line, with translocatable elements shown as coloured boxes or lines. The location of the acquired region in pT211 is indicated by a labelled horizontal line below, and identities of translocatable elements are indicated above the coloured boxes that represent them. The extent and orientation of named genes are indicated by labelled, horizontal arrows below. Drawn to scale from GenBank accession CP017083 B) Scaled, linear diagrams comparing the sequences of pT211 and cointegrate plasmid pT18 from T18. The sequence of pT211 is drawn as in part (A), and regions of pT18 derived from pT211 are indicated by grey shading between the plasmids. Sequence in pT18 derived from an FII-33 plasmid are shown as thin black lines, and sequence derived from pSCECL_a is shown as a pink line. The locations of antibiotic resistance genes in pT18 are indicated below, and the location of an additional 68 bp from Tn*2* in pT18 are indicated above. Drawn to scale from GenBank accessions CP017083 and CP017085.

The second plasmid in T21, pT211, is 24,225 bp, has an average G + C content of 54.4%, and contains a total of 30 ORFs. The 720 bp *repA* gene of pT211 differs by 3 nucleotides from the *repA* gene of the reference IncN plasmid R46 (GenBank accession AY046276), which was found in a *Salmonella enterica* isolated in the United Kingdom in 1962 [32]. Thus, pT211 is an N-type plasmid, but contains an incomplete, 12,972 bp N-type plasmid backbone (Figure 2A). The backbone lacks almost all of the conjugative transfer module of R46, but includes a putative *oriT* sequence that is 86% identical to the *oriT* of R46. Thus, pT211 is not expected to be conjugative.

The remaining 11,253 bp of pT211 is comprised of translocatable genetic elements, and includes *bla*_KPC-2_ (Figure 2A). This region is bounded on one end by IS*26* and by Tn*1722* on the other. The entire region is identical to the corresponding parts of the *bla*_KPC-2_-containing region of the F-type plasmid pKP048 (GenBank accession FJ628167) from a *K. pneumoniae* isolated in China in 2006. The *bla*_KPC-2_-containing region in pKP408 has been described previously [33].

### The *P. mirabilis* T18 plasmid pT18

The sole plasmid in T18, pT18, is 59,035 bp, has an average G + C content of 53.7% and contains 67 ORFs. It contains three replicons, and is a cointegrate plasmid comprised of sequences from three different plasmid backbones, one N-type, one F-type, and one rolling-circle type (Figure 2B; Table S4). Each of the regions of pT18 derived from the different progenitor plasmids are flanked by a pair of IS*26* (Figure 2B; Table S4).

The N-type plasmid region of pT18 is identical to the corresponding parts of pT211, and contains *bla*_KPC-2_ (Figure 2B). However, the pT211-derived region in pT18 is 24,293 bp, and the size discrepancy relative to pT211 can be explained by an IS*26*-mediated 68 bp deletion in pT211, as the additional bases in pT18 are immediately adjacent to IS*26* (Figure 2B; Table S4).

The F-type replicon in pT18 was typed as FII-33 using PubMLST. The regions adjacent to it also include the antibiotic resistance genes *bla*_CTX-M-65_, *bla*_TEM-1B_, *rmtB* and *fosA3* (fosfomycin resistance). These regions are identical to parts of the *E. coli* plasmid pHN7A8 (GenBank accession JN232517), which has been described previously [34]. However, parts of the FII-33 plasmid-derived regions in pT18 are shorter or inverted relative to pHN7A8 (Table S4), indicating that IS*26*-mediated inversion and deletion events have occurred in an intermediate plasmid that was the progenitor of pT18. The most significant region of pHN7A8 absent from pT18 is the transfer region, of which only the *traX* and *finO* genes remain adjacent to a copy of IS*26* (Table S4). On the opposite side of the IS*26* adjacent to *traX* is a 6,135 bp region that includes sequence from a truncated Tn*21* with a copy of IS*5075* inserted in IR_tnp_ (Figure 2B). This region separates sequences derived from the N and FII-33 plasmids, and is not present in pT211 or pHN7A8 (Table S4).

The final 2,886 bp of pT18 is comprised of a copy of IS*26*, an 8 bp target site duplication (see below) and 2,058 bp (pink in Figure 2B) from a small, rolling-circle plasmid identical to pSZECL_a (GenBank accession KU302803), a plasmid from an *Enterobacter cloacae* strain isolated in China in 2016. pSZECL_a is similar to the rolling-circle plasmid pBuzz from *E. coli* and contains two putative *oriT* sequences that resemble the *oriT* of L and M-type conjugative plasmids [35].

### Conjugative ability of pT211, pT212 and pT18

To test the conjugative ability of pT211 and pT212 from T21, and pT18 from T18, each *P. mirabilis* isolate was mated with the laboratory strain *E. coli* J53 in BHI broth. Consistent with the sequence data, which revealed that pT212 contains a complete transfer region, J53 transconjugants resistant to amikacin and sodium azide were obtained from a T21-J53 mating experiment. The presence of pT212 in transconjugants was confirmed by PCR. The conjugation frequency of pT212 was 7.47(±2.37)×10^−4^ transconjugants/recipient.

After three independent T21-J53 mating experiments, and three independent T18-J53 mating experiments, no transconjugants containing pT211 or pT18 were recovered. This is consistent with the sequence data of each of these plasmids.

### Effects of pT18 and pT211 on growth rate

**Figure 3.**
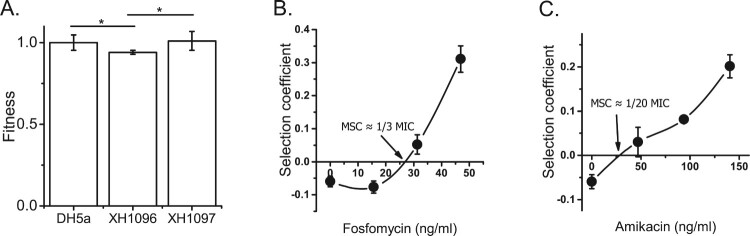
Effects of plasmids on DH5α growth rate. A) The growth rates of DH5α, XH1096 (DH5α pT18) and XH1097 (DH5α pT211) in MH medium. DH5α, XH1096 and XH1097 were grown in MH broth at 37 °C. The growth rates of the strains were determined by measuring the OD_600_ every 5 min and were estimated by an R Script based on the OD_600_ curves. Each strain represents four biological and three technical replicates. B) Competition experiments between susceptible and resistant strains (fosfomycin and amikacin). Competition experiments were performed at different concentrations of fosfomycin or amikacin, and selection coefficients were calculated as a function of antibiotic concentrations (B and C). The data represent the averages of three experiments Standard errors of the mean are indicated.

The fitness costs of pT18 and pT211 in a new host were determined by transferring them to laboratory *E. coli* DH5α by transformation. The growth rates of DH5α carrying pT18 or pT211 relative to wild-type DH5α were determined (Figure 3A). DH5α carrying pT211 grew at a similar rate to the wild type, while DH5α carrying pT18 grew at a reduced rate. This suggested that only the larger, cointegrate plasmid pT18 imposed a fitness cost on DH5α.

To evaluate the fitness cost of pT18 and pT211 in *P. mirabilis*, pT18 and pT211 were transformed into the plasmid-free *P. mirabilis* strain XH1568. The MICs of amikacin and fosfomycin for XH1568 and the XH1568 transformants containing pT211 (XH1570) or pT18 (XH1571) were determined using primers in Table S3. The growth rates of XH1568, XH1570 and XH1571 in MH broth (Figure 5 A), revealing that XH1568 grew at a similar rate with and without pT211, but grew slower when it contained pT18. This indicated that, as for *E. coli*, pT18 imposed a fitness cost on *P. mirabilis*.

### Plasmid pT18 provides a fitness benefit at low antibiotic concentrations in both *E. coli* and *P. mirabilis*.

To determine whether the observed fitness cost of pT18 was offset by the presence of antibiotics, DH5α carrying pT18 was competed against fluorescently marked DH5α in broth. First, control experiments demonstrated that carriage of the SYFP2-encoding fluorescence plasmid imposed only a slight cost (0.05) on DH5α, and that the SYFP2-encoding plasmid was stable in DH5α in the absence of antibiotic selective pressure (Figure S2).

As in the growth rate experiments, fluorescent DH5α grew faster than DH5α carrying pT18, outcompeting it (Figure 3A). However, as concentrations of fosfomycin or amikacin in broth increased, the apparent fitness cost of pT18 diminished, until the carriage of pT18 resulted in a fitness advantage (Figure 3B and C). The concentrations of fosfomycin or amikacin at which DH5α carrying pT18 outcompeted fluorescent DH5α were called the minimum selective concentration (MSC). For fosfomycin, the MSC (25 ng/ml) was 1/3 of the MIC for DH5α, and for amikacin, the MSC (34 ng/ml) was approximately 1/20 of the MIC for DH5α. Thus, the MSCs for both antibiotics were significantly lower than the MICs for DH5α.

**Figure 4.**
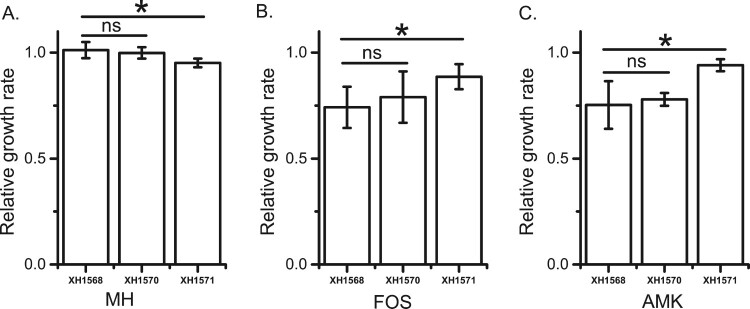
Effects of plasmids on *P. mirabilis* XH1568 growth rate. The growth rates of XH1568, XH1570 (XH1568 pT211) and XH1571 (XH1568 pT18) in A) MH medium, B) MH medium containing 32 mg/L fosfomycin, C) MH medium containing 4 mg/L amikacin. XH156, XH1570 and XH1571 were grown in MH broth with or without antibiotic at 37 °C. The growth rates of the strains were determined by measuring the OD_600_ every 5 min and were estimated by an R Script based on the OD_600_ curves. Each strain represents four biological and three technical replicates. The data represent the averages of three experiments Standard errors of the mean are indicated.

As we were unable to construct fluorescently-tagged *P. mirabilis* strains, we compared the growth rates of three *P. mirabilis* strains (XH1568, XH1570, and XH1571) in sub-MIC concentrations of amikacin and fosfomycin. *P. mirabilis* cells containing pT18 (XH1571) demonstrated a higher growth rate than wild type cells (XH1568) in 2 mg/L amikacin or 32 mg/L fosfomycin (1/2 MIC for XH1568) (Figure 4B, C, Table S5), suggesting that, as in *E. coli*, these plasmids provide a fitness advantage at sub-inhibitory antibiotic concentrations.

### The distribution of pT211 and pT18 in further *P. mirabilis* clinical isolates

**Figure 5.**
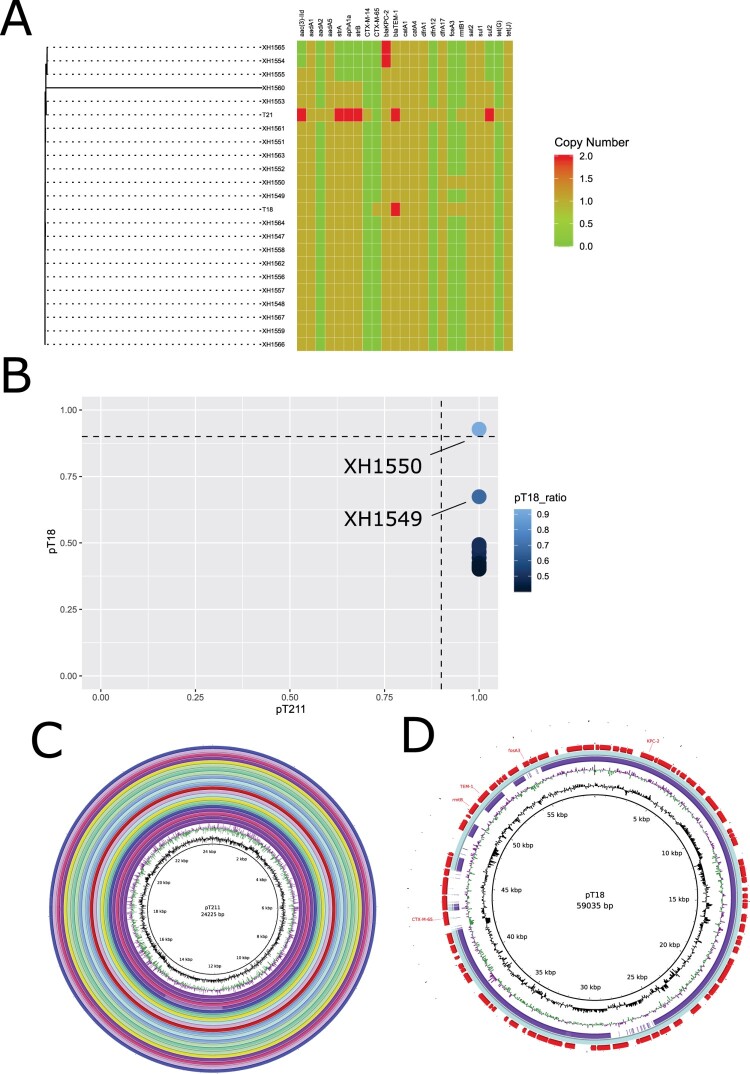
A) Maximum likelihood phylogenetic tree based on the 23 genome sequences from this study. Sequencing reads were mapped to *P. mirabilis* T21. The tree is based on 988 chromosomal SNPs. Branch lengths represent the number of SNPs. Antibiotic resistance genes are visualized in compliance to the tree. B) The plasmids coverage of pT211 and pT18 in 21 *Pmirabilis* clinical isolates. Plasmid coverage was identified by mapping illlumina reads (bwa 0.7.17-r1188) followed by samtools depth analysis. C) Alignment of pT211 identified in this study with contigs from twenty *P. mirabilis* isolates harboured pT211 plasmid. The map was constructed using BRIG software. D) Alignment of pT18 identified in this study with contigs from XH1549 and XH1550. The map was constructed using BRIG software. Light green: XH1550; purple: XH1549.

To determine whether pT211 and pT18 were widely distributed in *P. mirabilis* isolated in the same hospital at approximately the same time as T21 and T18, a further 21 *Pmirabilis* clinical isolates were sequenced on the Illumina platform. A phylogenetic tree was constructed using these isolates combined with the fully sequenced T18 and T21 genomes, showing the clonal nature of the collection (Figure 5A). Sequence reads of all isolates mapped to the complete pT211 sequence, suggesting that pT211 or close relatives are present in all of them. However, the FII-33 *repA1* gene was only detected in two isolate, XH1549 and XH1550. When XH1550 sequencing reads were mapped against pT18, they covered 92.8% of the pT18 sequence meanwhile XH1549 covered 67.4% of the pT18 sequence. Thus, of the additional 21 *Pmirabilis* isolates examined, only XH1549 and XH1550 contains an FII-33 replicon and contains a cointegrate plasmid closely related to pT18. Using BRIG, we visualized the presence of pT18 in XH1549 and XH1550 (Figure 5C) and pT211 in the other isolates (Figure 5D). In total, we found 21 isolates harboured pT211 plasmid and two of them (XH1549 and XH1550) carried pT18-like plasmids.

## Discussion

### Chromosomal resistance genes

*P. mirabilis* T18 and T21 have previously been shown to be clonal [5]. Consistent with this, the 15 antibiotic resistance genes found in the chromosomes of T18 and T21 are located in the same structures, and these confer resistance to several classes of antibiotics. Apart from the *cat* [36] and *tet*(J) [37] genes, which are native to *P. mirabilis*, the antibiotic resistance genes have been acquired as part of translocatable elements. Three resistance genes are part of Tn*7*, and the remaining 10 resistance genes were found in a complex resistance region derived from the insertion of a copy of a Tn*2670*-like transposon.

It appears that the original Tn*2670*-like transposon has been subject to a series of insertions and rearrangements leading to the complex structure present in T18 and T21. This situation is reminiscent of the antibiotic resistance islands of *Acinetobacter baumannii* global clone 1 [38,39], where, following an initial insertion into the chromosome, a complex island evolved, acquiring a series of antibiotic resistance determinants over several decades. It will be interesting to study the evolution of the resistance island in T18 and T21 by comparing it to related regions in future isolates of the same *P. mirabilis* clone.

### Divergent plasmid content of T18 and T21

In both T18 and T21, the chromosomal antibiotic resistance genes are supplemented by a set of plasmid-borne antibiotic resistance genes. Notably, the *bla*_KPC-2_ carbapenemase gene and the *rmtB* gene that confers resistance to all clinically relevant aminoglycosides are carried by plasmids. However, T18 and T21 carry different plasmids, indicative of independent but convergent evolutionary trajectories. In T21, the *bla*_KPC-2_ gene is in the N-type plasmid pT211, while the *rmtB* and other resistance genes are in the type 2 IncC plasmid pT212. In T18, *bla*_KPC-2_, *rmtB* and three more resistance genes are in the cointegrate plasmid pT18.

### Cointegrate plasmid pT18, IS*26* and horizontal gene transfer

The 59 kbp plasmid pT18 is clearly a cointegrate derived from three distinct plasmids; an N-type plasmid similar to pT211 of T21 (isolated in 2013), an FII-33 plasmid similar to pHN7A8 from an *E. coli* isolated in China in 2008, and the rolling-circle plasmid pSZECL_a from an *Enterobacter cloacae* isolated in China in 2012. The precise series of events that led to the formation of such a cointegrate cannot be determined in the absence of complete sequences of all evolutionary intermediates that existed between ancestral plasmids and the plasmids that formed pT18. However, to form a cointegrate, parental plasmids must be present in the same bacterial population at the same time. The presence of a cointegrate comprised of plasmids related to examples originally found in three different genera is strong evidence for the horizontal transfer of these plasmids between these members of the Enterobacterales. As plasmids related to all three proposed parental plasmids were found in China within a 5-year period, the cointegration events that generated pT18 likely occurred in China, but the environment or bacterial host in which they occurred cannot be determined using the data that are currently available.

**Figure 6.**
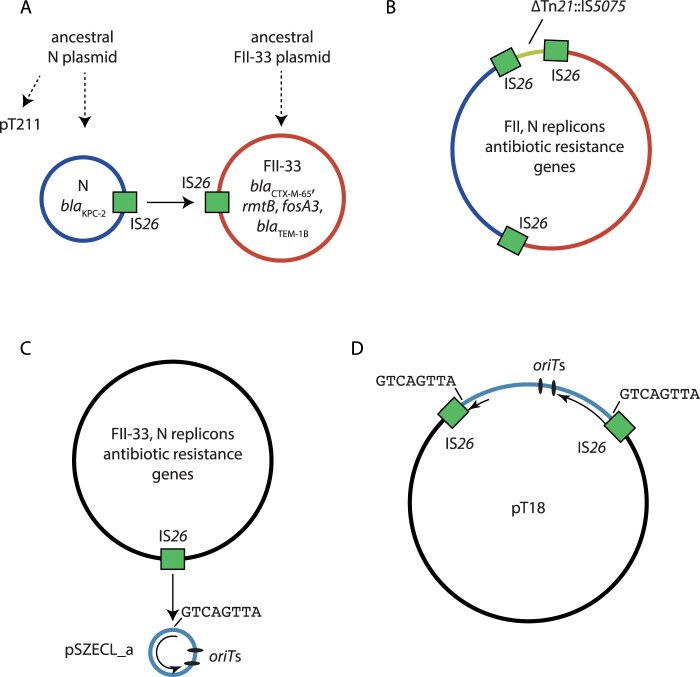
Proposed mechanism for the formation of cointegrate plasmid pT18. Schematic showing A) progenitor N (red) and FII-33 (blue) plasmids that formed a cointegrate to generate B) a cointegrate containing N and FII-33 plasmid sequences via a conservative IS*26* transposition event (indicated by a solid arrow). IS*26* involved in the event are shown as green boxes, and the positions of 8 bp sequences adjacent to each IS*26* are indicated by black lines. Evolution of proposed progenitor plasmids from ancestral plasmids via insertions, deletions or inversions are indicated by broken arrows. Part C) shows the progenitor N/FII-33 (black) and pSZECL_a (pale blue) plasmids that formed a cointegrate to generate D) pT18 via a replicate IS*26* transposition event. The single IS*26* involved in the event is shown as a green box in C), and the resultant pair of IS*26* are shown as green boxes in D). The position of the 8 bp sequence at the IS*26* insertion site in pSZECL_a is indicated by a black line in C), and the positions of the duplicated sequence are indicated in D). The extent of the pSZECL_a *rep* gene is shown as a black arrow, and the positions of putative *oriT* sites in pSZECL_a are shown as labelled black ovals.

From analysis of available sequence data, we hypothesize that pT18 was formed by at least two IS*26*-mediated reactions, but the exact order of these events cannot be determined. A cointegrate formed from the progenitor N and FII-33 plasmids was likely generated by a conservative IS*26* transposition event (Figure 6A), as copies of IS*26* are present in plasmids (pT211 and pHN7A8) closely related to the pT18 parent plasmids. This event would have involved an IS*26* in each of the progenitor DNA molecules, and would not yield any additional copies of IS*26* (Figure 6B), or a target site duplication. Acquisition of the Tn*21*::IS*5075*-containing region (yellow in Figure 3B) flanked by IS*26* could have occurred in a parental plasmid or after cointegration, but this cannot be determined with the available sequence data. The incorporation of rolling-circle plasmid pSZECL_a likely involved replicative transposition of IS*26*. In this case, one of the IS*26* copies in either the N-type/FII-33 cointegrate or either progenitor plasmid transposed into the small plasmid (Figure 6C). This event generated an additional copy of IS*26* plus an 8 bp target site duplication in the integrated pSZECL_a (Figure 6D). As the IS*26* transposed into the *rep* gene of pSZECL_a, it is no longer capable of initiating replication, and exists as passenger DNA in pT18.

Based on our analyses, pT18 represents another example of IS*26*-mediated convergence of antibiotic resistance determinants, adding to existing evidence for the importance of IS*26* and other members of the same IS family in mobilizing antibiotic resistance genes in both Gram-negative and Gram-positive bacteria [40]. In addition, it appears that in the case of pT18 IS*26* may also have contributed to intercellular mobility of antibiotic resistance genes, by integrating the rolling-circle plasmid pSZECL_a, which contains two putative *oriT* sites [35]. Neither of pT211 or pT18 are conjugative, as the N-type plasmid backbone they share lacks the complete set of genes required for transfer. The transfer region of the FII-33 backbone in pT18 is also incomplete. However, pT18 includes two *oriT* sequences that are not found in pT211 or pHN7A8. If the *oriT*-like sequences in the pSZECL_a portion of pT18 can be recognized by co-resident L or M-type conjugative plasmids, which have been seen in *P. mirabilis* [41], it is possible that pT18 can be mobilized by a relaxase-*in trans* mechanism [9].

### Effects of plasmids on host growth rate

An interesting outcome of this study was the finding that pT18, but not pT211, imposed a fitness cost on *E. coli* and *P. mirabilis*. The mechanisms that contribute to plasmid fitness cost are not well understand [42], and therefore it is difficult to determine why pT18 imposes a fitness cost. It is possible that the size of pT18, the number of antibiotic resistance genes it carries, or the presence of an additional plasmid replicon might play a role. The fitness costs of diverse plasmids have previously been studied by a combination of phenomics, transcriptomics and metabolomics in *Pseudomonas aeruginosa* PAO1. It was shown that fitness cost of plasmids had a complex origin, and the plasmid changed the expression of a common set of metabolic genes [43]. The effects of various plasmid characteristics on plasmid fitness cost in *P. mirabilis* still remain to be investigated.

It will also be interesting to determine whether any particular selective pressure selected for the cointegration of FII-33 and N-type plasmids in *P. mirabilis*. As pT211 was found in *P. mirabilis* T21, N-type plasmids appear to replicate stably in this host. In contrast, the FII-33 replicon in pT18 has not been reported in *P. mirabilis* before, and a recent search of the GenBank non-redundant nucleotide database revealed that this replicon was only present in plasmids from *E. coli* and *K. pneumoniae* (last searched September 12, 2019). Thus, FII-33 plasmids might not replicate stably in *P. mirabilis*, and upon entry to a *P. mirabilis* cell, could only be maintained following cointegration with a more stable N-type plasmid. The stability of FII-33 and N-type plasmids in *P. mirabilis* will thus require further investigation.

### Selection for plasmid carriage at sub-inhibitory antibiotic concentrations

Despite the observed fitness cost of pT18 to DH5α and *P. mirabilis* in non-selective media, competition experiments showed that pT18 could be selected for with concentrations of fosfomycin and amikacin far below the DH5α MIC of either antibiotic. While antibiotic concentrations in natural environments can be highly variable[44], these findings highlight the potential for multiple low level antibiotics in the natural environment or clinical context to select for MDR plasmids.

## Conclusions

In both *P. mirabilis* isolates examined here, *bla*_KPC-2_ was found in an N-type plasmid, but in one isolate the N-type plasmid was part of a cointegrate that also contained sequence derived from FII-33 and rolling-circle plasmids. The cointegrate appears to have been generated by the actions IS*26*, emphasizing its importance in the accumulation of antibiotic resistance genes in Enterobacterales plasmids. This study raises questions about the role of cointegrate plasmids in the dissemination of antibiotic resistance genes between pathogenic bacterial species, and highlights the importance of sub-inhibitory environmental concentrations of antibiotics to the persistence of such plasmids.

## Data Availability

The sequence data for the chromosomes and plasmids of T21 and T18 have been deposited in GenBank under the accession numbers CP017082-CP017086. The whole genome sequences of XH1547-XH1567 have been deposited in GenBank under accession numbers JAAONO000000000 – JAAOMU000000000. The complete genome sequence of XH1568 has been deposited in GenBank under accession number CP049941.
